# Neuroendoscopic treatment of multiple intracranial arachnoid cysts: a case report

**DOI:** 10.1186/s41016-018-0124-9

**Published:** 2018-07-04

**Authors:** Di Chen, Jun Zhang, Lixin Wu, Xueyuan Li, Siqi Ma, Xuqiang Zhu, Dongming Yan

**Affiliations:** 1 Department of Neurosurgery, The First Affiliated Hospital of Zhengzhou University, Zhengzhou, 450052 China; 2 Department of Orthopedics, Zhengzhou Orthopedics Hospital, Zhengzhou, 450052 China

**Keywords:** Arachnoid cysts, Endoscopic fenestration, Obstructive hydrocephalus

## Abstract

**Background:**

Multiple arachnoid cysts are very rare within the central nervous system. The cysts will sometimes increase in size with age, lead to the mass effect or cerebrospinal fluid (CSF) flow obstruction, and cause some symptoms, which requires the surgery intervention.

**Case presentation:**

A 35-year-old female was admitted to our hospital with some symptoms related to hydrocephalus for 1 month. Brain magnetic resonance imaging (MRI) revealed well-marginated cystic lesions with CSF signal intensity in the ventricular and cisternal systems, bilateral temporal lobes, and left occipital lobe. Cine phase-contrast MRI showed the aqueduct of sylvius was blocked by the cyst in the quadrigeminal cistern. We employed endoscopic ventriculocystostomy and septostomy to create the communication of the cyst with ventricular and cistern system. As a result, the patient’s symptoms were relieved.

**Conclusions:**

Endoscopic management can be an effective way for treating intracranial multiple arachnoid cysts, which is the first report of this kind. We hope that this report could help improve the management of intracranial arachnoid cysts with the neuroendoscopic technology.

## Background

Arachnoid cysts are fluid-collected intracranial lesions that can be located in different parts of the cranial cavity. Most arachnoid cysts are congenital, clinically silent, and remain stable in size. Occasionally, they will increase in size and cause some symptoms due to mass effect or the cerebrospinal fluid (CSF) flow obstruction. The most common symptoms include headache, nausea, vomiting and visual impairment. When the lesions cause symptoms, surgery must be performed. More and more neurosurgeons regard endoscopic therapy as the optimal choice of cranial arachnoid cysts due to minimal invasion, fewer complications, cheaper treatment costs and shorter hospital stay [1, 2]. This report will introduce an innovative management of multiple intracranial arachnoid cysts with the help of neuroendoscopy.

## Case presentation

**Fig. 1:**
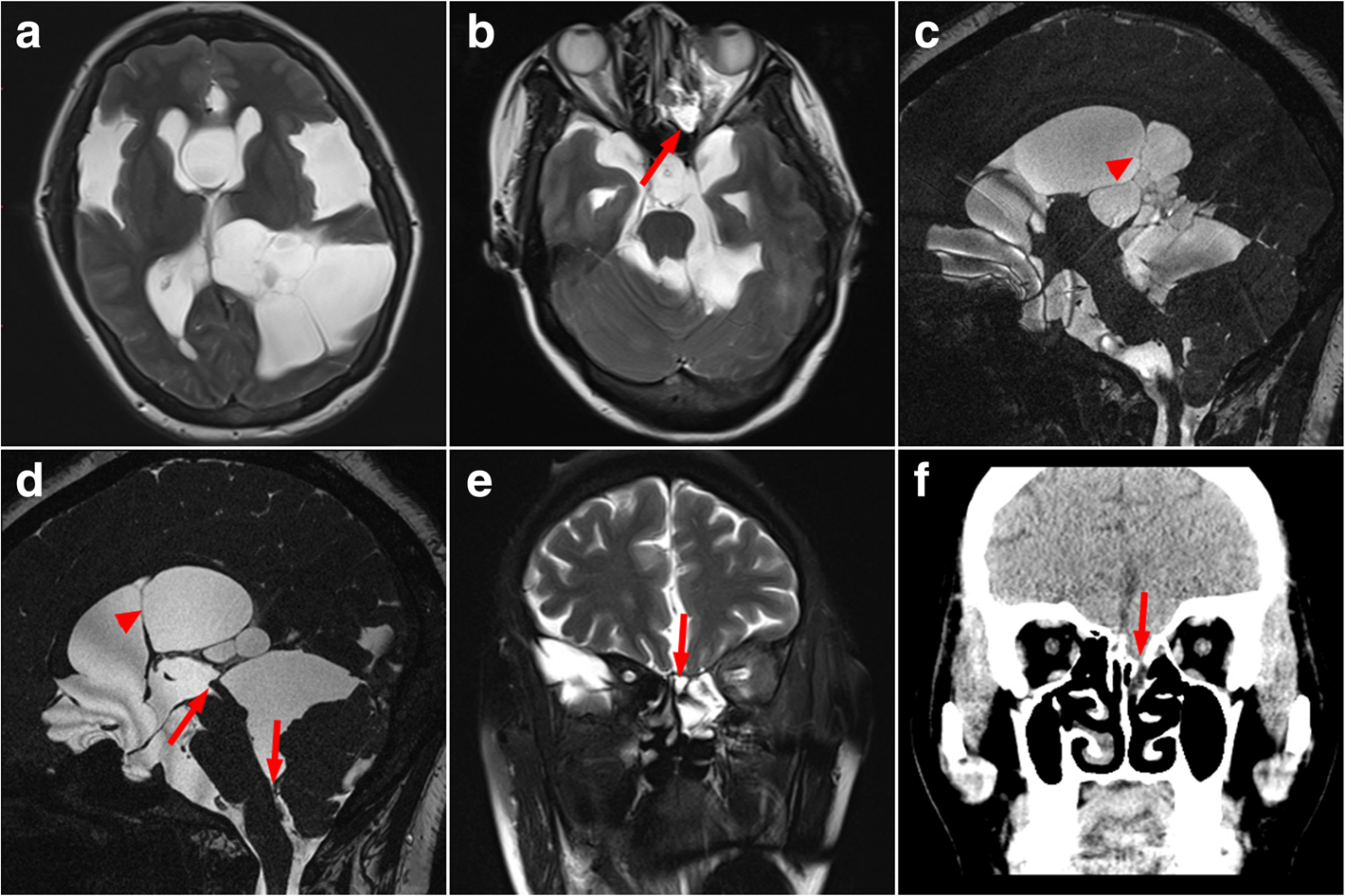
Preoperative MR image. **a** and **b** The T2-weighted axial image: multiple arachnoid cysts. **c** and **d** Preoperative Cine phase-contrast MR image: Arrow directs that the aqueduct of sylvius and the exit of fourth ventricle are blocked. **e** The T2-weighted coronal MR image: Arrow shows that the left anterior cranial fossa is likely communicated with the left ethmoid sinus. **f** A coronal high-resolution bone window noncontrast CT image: Arrow indicates that left ethmoid is discontinuous

A 35-year-old female was admitted to our hospital with a constellation of symptoms related to hydrocephalus for 1 month, including progressive headache and dizziness, nausea, vomiting, and visual impairment. Brain magnetic resonance imaging (MRI) revealed well-marginated CSF signal intensity cystic lesions located in bilateral temporal lobes, left occipital lobe, ventricular and cisternal systems. The prepontine cistern, interpeduncular cistern, suprasellar cistern, quadrigeminal cistern, cavum septum pellucidum and third and lateral ventricles were markedly dilated (Fig. 1a and b). Cine phase-contrast MRI showed the aqueduct of sylvius was blocked by cyst originated from quadrigeminal cistern (Fig. 1c and d). A high-resolution bone window noncontrast CT image indicates that left ethmoid is discontinuous (Fig. 1f). Furthermore, intracranial pressure was determined to be as high as 510 mmH_2_O with a lumbar puncture.

**Fig. 2:**
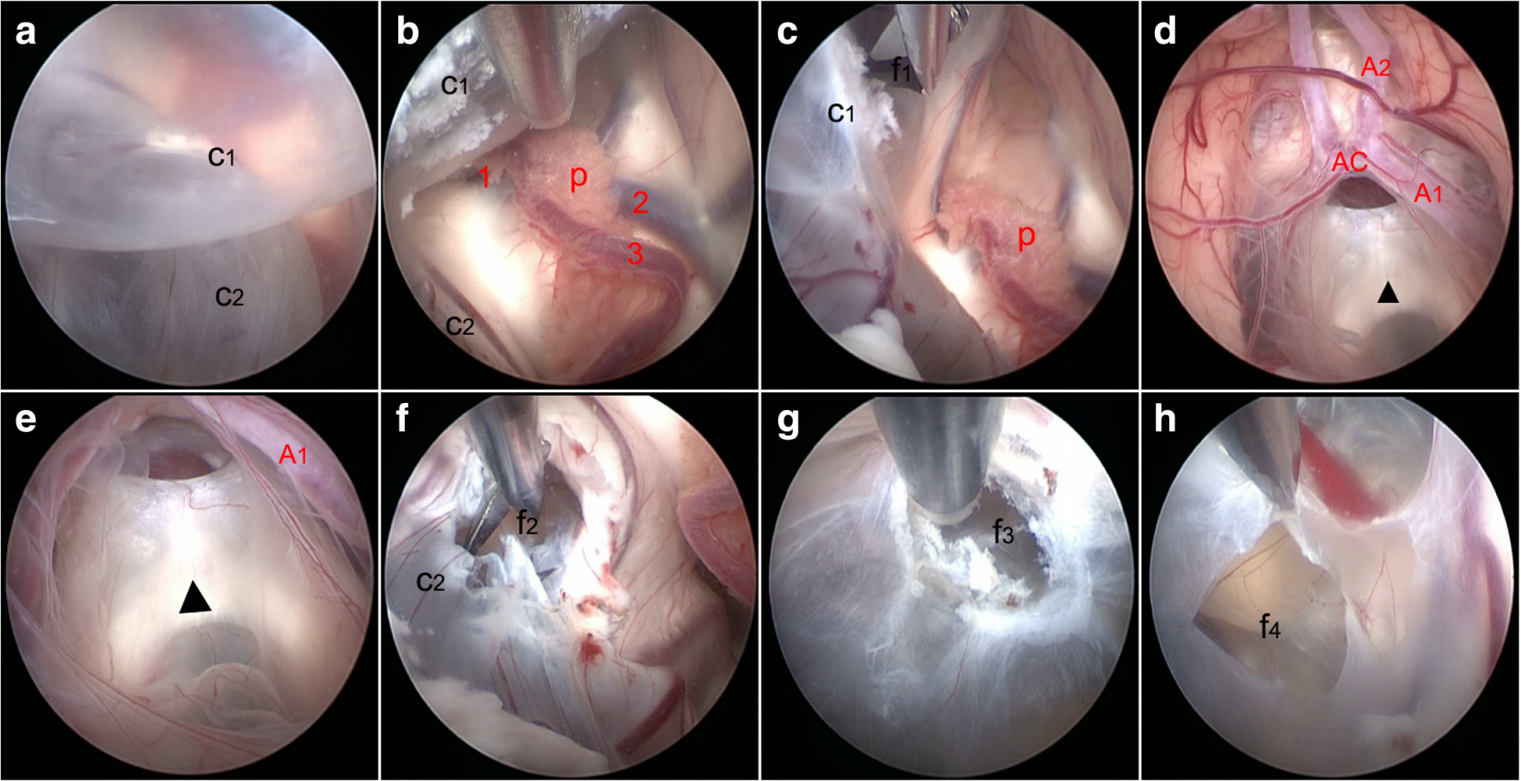
Intraoperative neuroendoscopic views. **a** Two cysts (c_1_ and c_2_) bulging into the right lateral ventricle. **b** Along the choroid plexus we find no trace of the foramen of Monro. **c**: Fenestration of the apical membrane in front of the midline (f_1_). **d** and **e** Anatomical landmarks seen after the endoscope is advanced into the first cyst (c_1_). **f** Fenestration of the apical membrane behind the midline (f_2_). **g** and **h** Two fenestrations of the basal cyst membrane behind the midline (f_3_ and f_4_). p = plexus choroideus; 1 = septal vein; 2 = thalamostriate vein; 3 = choroidal vein; A_1_ = the horizontal segment of the anterior cerebral artery; A_2_ = the ascending part of the anterior cerebral artery; AC = anterior communicating artery; ▲Indicates the optic chiasma

We used endoscopic ventriculocystostomy and septostomy to establish a new communication of the cyst with the ventricular or cistern system, with a purpose of releasing the intracranial pressure and relieving the symptoms. Under general anesthesia, the patient was in the prone position. After a 10-cm arc scalp incision was made, a 3*2 cm free small bone flap about 2.5 cm lateral to the midline and 1 cm anterior to the coronal suture was created. After the dural opening and exposure of the right cerebellar hemisphere, we used a Cushing puncture needle and a rigid endoscope with 6° lens was inserted through this puncture tract. In the right lateral ventricle, we found the walls of two cysts (Fig. 2a). However, we did not find the foramen of Monro along the choroid plexus. We speculated that the foramen of Monro was obstructed by the membrane of a cyst. First, we burn the cyst wall in front of the midline to make it shrivel. Then, the cyst wall was resected as much as possible by using scissors to make a large fenestration (f_1_) in its apical membrane (Fig. 2b and c). Behind the fenestration, some anatomical landmarks such as chiasmatic cisterns, anterior cerebral artery, optic chiasma were clearly visualized (Fig. 2d and e). After inspection, the endoscope was further advanced to the basal cyst membrane. We found that part of the basal cyst membrane was missing and CSF circulates smoothly. Therefore, the endoscopy was retracted into the ventricle. Then, we make a fenestration in avascular portions of the apical cyst membrane behind the midline by using scissors (Fig. 2f). After fenestration, there were several cyst walls like the silkworm cocoon contacting each other in the floor of this cyst and we burnt and resected these cyst walls. Finally, we make two fenestrations in the basal cyst membrane, which would allow communication between the cyst and quadrigeminal cistern (Fig. 2g and h). After retraction of the endoscope, the cortical tract was plugged with gelatin sponge. The dura was sealed and then the subcutaneous and skin.

**Fig. 3:**
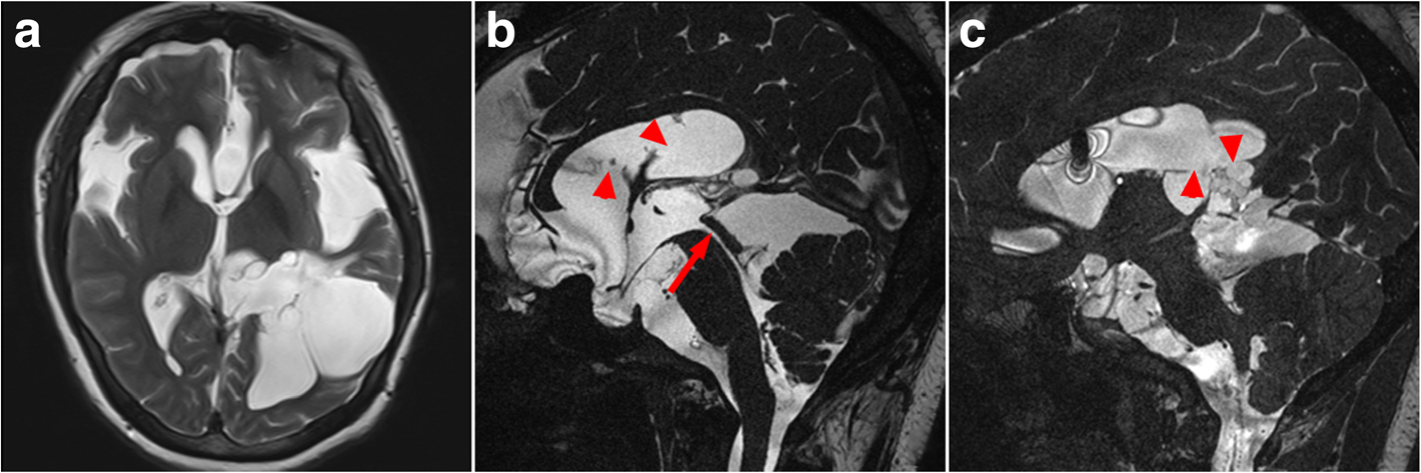
Images obtained a week after the operation. **a** T2-weighted axial MR image. **b** and **c** Cine phase-contrast MR image. **b** Arrow indicates that satisfactory CSF flow through aqueduct of Sylvius is detected. ▲Indicates the fenestrations of the apical membrane (f_1_ and f_2_). **c** ▲Indicates two fenestrations of the basal cyst membrane behind the midline (f_3_ and f_4_)

**Fig. 4:**
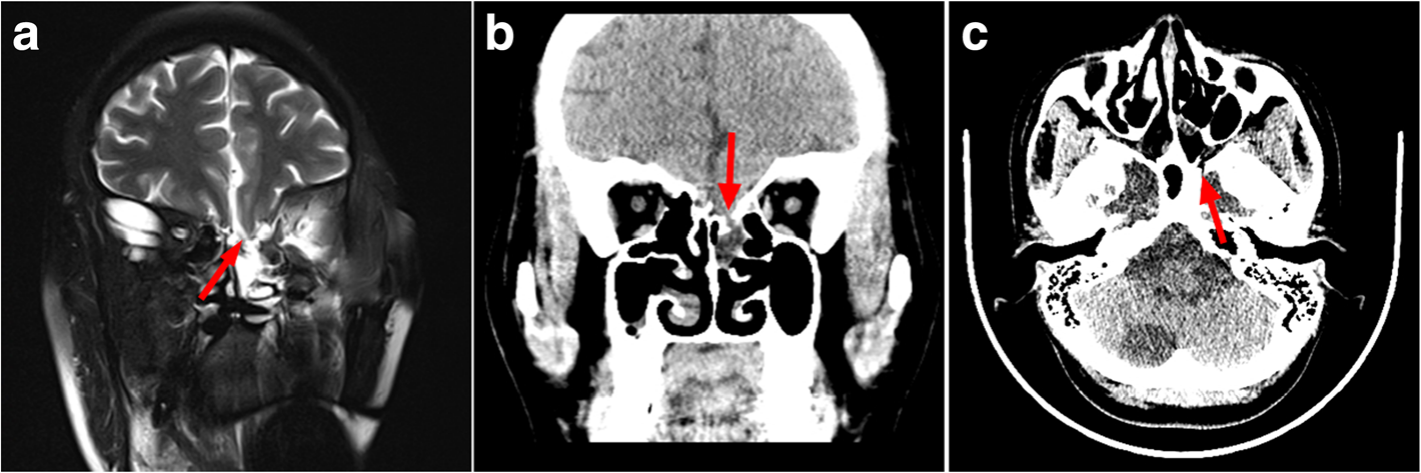
Images obtained 9 months after the operation. **a** T2-weighted coronal MR image. Arrow shows that the left anterior cranial fossa is communicated with the left ethmoid sinus. **b** and **c** The coronal and axial high-resolution bone window noncontrast CT images: Arrow indicates that there is a poor continuity of bone in the left ethmoid

Headache and dizziness, nausea and vomiting disappeared, and visual acuity improved slightly with no adverse postoperative reaction. Postoperative MRI and lumbar puncture were done after a week and 6 months respectively, and the shape of fourth ventricle was back to normal and satisfactory CSF flow through aqueduct of Sylvius was detected. MRI displayed that the volume of lateral ventricles and the cavity of septum pellucidum decreased well (Fig. 3). The intracranial pressure was 210 mmH_2_O 6 days after surgery, and 300 mmH_2_O 6 months after surgery. These findings indicated a partial resolution of hydrocephalus. 9 months later, however, the patient developed some symptoms of spontaneous cerebrospinal fluid rhinorrhea and uncontinuous fever. MRI showed that the left anterior cranial fossa was communicated with the left ethmoid sinus and the computed tomography (CT) indicated that there is a poor continuity of bone in the left ethmoid (Fig. 4). The lumbar puncture pressure was 260 mmH_2_O and the CSF routine biochemical tests showed that the number of nuclear cells (61 × 10^6^/L) and total protein (904 mg/L) was elevated, and that glucose (1.73 mmol/L) and chlorine (118 mmol/L) was decreased. Considering the patient’s CSF rhinorrhea was intermittent and not serious, and she had a fever, the operation was not performed to repair the leakage. Three generations of cephalosporin antibiotics and absolute bed rest were treated to the patient. Two weeks later, the symptoms of CSF rhinorrhea and fever disappeared. The lumbar puncture CSF revealed that the number of nuclear cells, total protein, glucose and chlorine were within normal range. After the patient was discharged from the hospital, we advised her to stay in bed for another month and avoid a severe sneeze. During the two years of follow-up from her last discharge, the patient was in a good condition.

## Discussion and conclusions

Arachnoid cysts are assumed to be congenital lesions developing at various locations in the central nervous system [2]. In a study, a total of 48,417 adults underwent brain MRI examination and arachnoid cysts were identified in 661 patients (1.4%), multiple arachnoid cysts occurred only in 30 patients (0.062%) [3]. Multiple arachnoid cysts are exceedingly rare, and we achieve a good result with an endoscope to treat a patient with so many cysts and walls.

The causes of these cysts are always being under debate, and the natural history of any particular arachnoid cyst is unknown. Most arachnoid cysts are stable and clinically silent, some have the potential to progressively enlarge, but on rare occasions they disappear spontaneously [4]. The mechanisms behind cyst development are still poorly understood. Many hypotheses, such as active secretion of CSF by the cyst wall [5], a slit-valve mechanism [6], an osmotic gradient between the cystic content and the CSF [7], have been proposed. However, none of them could perfectly explain the cause of intracranial arachnoid cysts including multiple cysts.

However, the patient had intermittent spontaneous cerebrospinal fluid rhinorrhea 9 months after surgery, and we think three reasons can explain the situation: 1.the patient had natural fluctuations in intracranial hypertension that resulted in periodic pressure shifts that were crucial enough to develop a skull base erosion at sites of inherent structural weakness [8]. From the preoperative high-resolution bone window noncontrast CT image, we can see that left ethmoid is discontinuous (Fig. 1f). 2. Arachnoid cysts in proximity to the ethmoid and sphenoid sinus had expanded gradually with pulsatile-increased intracranial pressure, which have been implicated as precursors of the erosion of the dura of the sellar diaphragm [9]. From the preoperative T2-weighted coronal MR image, we can know that the left anterior cranial fossa is likely communicated with the left ethmoid sinus. 3. The changes of intracranial pressure after the operation caused the cerebrospinal fluid to flow out of the erosion of the dura and ethmoid, which leads to cerebrospinal fluid rhinorrhea.

With the continuous development of neuroendoscopic technology, more and more surgeons regard endoscopy as the first choice to treat arachnoid cysts [2, 3]. Usually, more anatomical structures can be seen and the endoscopic procedures are easier to perform than other ways in cases of the arachnoid cysts because the space is big enough for movement of the endoscope and instruments (Fig. 2). Endoscopic management has been proven to be effective, safe and have fewer complications [1]. The average length of staying in hospital is significantly reduced, and patients can resume their ordinary activities soon. Endoscopic techniques have been repeatedly shown to be effective and safe in treating convexity, middle fossa, interhemispheric, intraparenchymal, Sylvian, quadrigeminal, and suprasellar arachnoid cysts [1, 2]. Therefore, the neuroendoscopic technology is a good choice for the patients of intracranial multiple arachnoid cysts.

Multiple arachnoid cysts are exceedingly rare, and this is the first case of many cysts treated with endoscopy. We believe that endoscopic management can be an effective way to treat multiple arachnoid cysts.

## Abbreviations

CSF: Cerebrospinal fluid
CT: Computed tomography
MRI: Magnetic resonance imaging
